# Editorial: Analytical chemistry applied to natural products: trends and challenges

**DOI:** 10.3389/fphar.2023.1235224

**Published:** 2023-07-04

**Authors:** Herbert Júnior Dias, Caio Pinho Fernandes, Hidayat Hussain

**Affiliations:** ^1^ Núcleo de Química, Instituto Federal de Educação, Ciência e Tecnologia Goiano—Campus Urutaí, Urutaí, Goiás, Brazil; ^2^ Laboratório de Nanobiotecnologia Fitofarmacêutica, Universidade Federal do Amapá, Macapá, Amapá, Brazil; ^3^ Leibniz Institute of Plant Biochemistry, Halle, Germany

**Keywords:** natural products, dereplication, analytical method (AM), hyphenated techniques, bioprospeccion, traditional herbs, etnopharmacology

The chemistry of natural products has been used by humans since the beginning for various applications, particularly for obtaining isolated substances or fractions of compounds that can be used to produce natural remedies (Wangchuk, 2018). Consequently, the chemistry of natural products has become an important area for the technological development of new drugs, which are essential for society. Recent studies on natural products have estimated that in the last 40 years, around 23.5% of drugs approved worldwide have come from natural products or their derivatives (Newman and Cragg, 2020) Therefore, the identification of the chemical constitution of fractions of natural products (NP), isolated molecules of NP or semi-synthetic derivatives of these is a necessary factor for obtaining substances of commercial interest.

In this sense, the development of suitable analytical methods for qualitative or quantitative analysis of classes of metabolites obtained from NPs is essential for technological development in areas such as bioprospecting (Pushpangadan et al., 2018). Plants used in ethnopharmacology in different parts of the world are frequently used as models to identify prominent biological activities. Traditional Chinese Medicine, for example, has millenary origins and presents many interesting studies that aim to botanically identify medicinal plants, and establish correlations between the structure and pharmacological activity of classes of compounds obtained from these plants. Plants such as *Prunella vulgaris* L., described in the review by Pan et al. (2023) are an invaluable source of several classes of identified compounds, such as ursolic, oleanoic, linoleic, and arachidic acids, among others, which are responsible for various biological activities described in the literature for this plant.

Traditional Chinese medicine plays an important role in the control and regulation of antitumor processes using compounds identified in herbs common to local ethnopharmacology. The review described by Zhu et al. demonstrates that DNA methylations caused by epigenic drugs derived from Chinese herbs have the potential to develop reliable drugs against a wide range of tumors. Traditional Indian medicine also stands out in its action against various of known diseases. Studies that aim to identify substances from NPs that present neuroprotective activities have gained prominence, since these neurological disorders are often associated with the elderly, who are apt to use herbal medicines. Narouzkhani et al. synthesized various data on substances or organic fractions identified in Ayurveda, an alternative medicine system used for thousands of years in India.

Some plant species produce important NPs, studied and known all over the world (Calixto, 2019). One of the most important species that has natural products used against infectious diseases is the genus *Piper*, as described in the work by Vásquez-Ocmín et al. The authors describe the use of structural elucidation techniques such as liquid chromatography coupled with mass spectrometry (LC-MS) to identify chromatographic profiles of extracts from plants of the genus *Piper* that act as inhibitors of bacteria, fungi, and parasites without prior isolation of the molecules present in the NPs. Such techniques represent a breakthrough in the way metabolomic analysis is conducted in search of new bioactive compounds (Lopes and Silva, 2023). Another important genus common to traditional Chinese medicine and used all over the world is *Hedysarum*, from which important flavonoids, saponins, and alkaloids are identified, that demonstrate important applications against diabetes, cardiovascular diseases, Alzheimer’s disease and are also used as inflammation inhibitors, according to the mechanisms and clinical applications described by Gao et al.

In addition, the species *Scutellariae radix*, which is used in traditional Chinese medicine for anti-inflammatory purposes, as well as many other known ones, can be used according to the seasonality of the species for other pharmacological purposes. Such differences in the metabolites produced at different times of the year are a known fact in the literature, as well as the repositioning of traditional plants for other purposes, as described in the work of Han et al. In this study, the authors developed a database using liquid chromatography coupled to sequential mass spectrometry (LC-MS/MS) to identify active compounds against septicemia. Through studies using hyphenated techniques in combination with molecular modeling studies, oroxylin A was defined as a possible antiseptic agent, helping to identify the inhibition pathway of the biological receptor. Hyphenated techniques, similar to the one used in the work described above, have been widely used for the identification of pharmacokinetic aspects of known NPs (Singh et al., 2021), as in the study developed by Wei et al. Here, the authors identified molecules that act as biochemical markers in cardiovascular diseases using ultrahigh-performance liquid chromatography coupled to high-resolution Q-Extractive mass spectrometry (UHPLC-Q-Extractive HRMS) after treating living models with Xinkeshu, a Chinese patented drug formed by the combination of several NPs.

It is also important note that the discovery of new natural products has gradually evolved using efficient analytical methods to identify compounds with great biological potential in marine organism metabolism. Studies such as Guo and Wang, which describe a systematic review on the identification, pharmacological aspects, and mechanisms of action of glyceroglycolipids in marine algae, have been highlighted for their ability to detect compounds at minute concentrations in complex samples. Nevertheless, it is clear that the chemistry of marine NPs is a broad, yet insufficiently explored, area that is becoming more accessible due to the evolution of analytical methods. Analytical methods for the identification of natural products, both of marine and plant origin, have been constantly advancing and aiding the progress of medicinal chemistry. It is a multidisciplinary field, which often reviews an immense variety of new compounds that could potentially contribute to traditional medicine, as well as to other related fields such as the environment, human health in general, materials, and more. In face of current complex climatic changes, one of the most active methods for the conservation of NPs and the development of science is the evolution of analytical methods.

## Fundings

HD would like to thank Pró-Reitoria de Pesquisa, Pós-Graduação e Inovação—PROPPI-IFGoiano (grant number 23216.000929.2022-22) for the financial support. CF would like to thank Conselho Nacional de Desenvolvimento Científico e Tecnológico (CNPq) (grant number 317405/2021-7) for the financial support.
